# Accuracy of best possible medication histories by pharmacy students: an observational study

**DOI:** 10.1007/s11096-022-01516-2

**Published:** 2022-12-14

**Authors:** Martina Francis, Louise Deep, Carl R. Schneider, Rebekah J. Moles, Asad E. Patanwala, Linda L. Do, Russell Levy, Garry Soo, Rosemary Burke, Jonathan Penm

**Affiliations:** 1 Department of Pharmacy, Royal Prince Alfred Hospital, Camperdown, NSW Australia; 2 Faculty of Medicine and Health, School of Pharmacy, The University of Sydney, Camperdown, NSW Australia; 3 Department of Pharmacy, Concord Repatriation Geriatric Hospital, Concord, NSW Australia; 4 Department of Pharmacy, Prince of Wales Hospital, Randwick, NSW Australia

**Keywords:** Best possible medication history, Hospital admission, Medication discrepancy, Medication reconciliation, Pharmacy students, Transition of care

## Abstract

**Background:**

Medication reconciliation is an effective strategy to prevent medication errors upon hospital admission and requires obtaining a patient’s best possible mediation history (BPMH). However, obtaining a BPMH is time-consuming and pharmacy students may assist pharmacists in this task.

**Aim:**

To evaluate the proportion of patients who have an accurate BPMH from the pharmacy student-obtained BPMH compared to the pharmacist-obtained BPMH.

**Method:**

Twelve final-year pharmacy students were trained to obtain BPMHs upon admission at 2 tertiary hospitals and worked in pairs. Each student pair completed one 8-h shift each week for 8 weeks. Students obtained BPMHs for patients taking 5 or more medications. A pharmacist then independently obtained and checked the student BPMH from the same patient for accuracy. Deviations were determined between student-obtained and pharmacist-obtained BMPH. An accurate BPMH was defined as only having no-or-low risk medication deviations.

**Results:**

The pharmacy students took BPMHs for 91 patients. Of these, 65 patients (71.4%) had an accurate BPMH. Of the 1170 medications included in patients’ BPMH, 1118 (95.6%) were deemed accurate. For the student-obtained BPMHs, they were more likely to be accurate for patients who were older (OR 1.04; 95% CI 1.03–1.06; *p* < 0.001), had fewer medications (OR 0.85; 95% CI 0.75–0.97; *p* = 0.02), and if students used two source types (administration and supplier) to obtain the BPMH (OR 1.65; 95% CI 1.09–2.50; *p* = 0.02).

**Conclusion:**

It is suitable for final-year pharmacy students to be incorporated into the BPMHs process and for their BPMHs to be verified for accuracy by a pharmacist.

**Supplementary Information:**

The online version contains supplementary material available at 10.1007/s11096-022-01516-2.

## Impact statements

- This is the first study evaluating the accuracy of pharmacy student-obtained best possible medication histories when subsequently verified by a pharmacist.
- The results from this study suggest that final year pharmacy-students may be used as a time-efficient resource to support pharmacists in obtaining best possible medication histories upon hospital admission.
- This study assists hospital and educational institutions to educate and employ final-year pharmacy students to support medication continuity across transition of care.

## Introduction

Transitions of care, such as hospital admission and discharge, have been identified as vulnerable points for patient safety because of the high risk of error due to a lack of continuity between healthcare professionals and settings [1]. Medication continuity is one aspect that compromises patient safety, with an estimated 60% of medication errors occurring during transition of care [1]. The World Health Organisation reports that up to 97% of adult patients had at least one medication discrepancy specifically on admission to hospital [2], and almost half of these medication discrepancies are considered serious [3]. Overall, medication-related adverse drug events (ADE) are now estimated to be the 14th leading cause of morbidity and mortality globally, putting patient harm in the same league as tuberculosis and malaria [2].

In the hospital setting, medication reconciliation is considered an effective strategy to prevent medication errors upon admission [2]. Medication reconciliation upon hospital admission refers to the process in which medications that should be prescribed match those that are prescribed [1, 2, 4]. A recent meta-analysis has shown that pharmacist-led medication reconciliation programmes led to a reduction of 67% in ADE-related hospital revisits; 28% in ED visits; and 19% in all-cause hospital readmissions [5]. When conducting a medication reconciliation, the first step is to obtain a Best Possible Medication History (BPMH), which can be time-consuming [6]. This is due to BPMHs, ideally, being obtained by using multiple sources of information to ensure its accuracy. Such sources include a community pharmacy dispensing history or a general practitioner’s (GP) medication list [7]. The second step is to compare the patient’s BPMH to their inpatient medication list and appropriately rectify any identified deviations [7].

Pharmacists have been identified as the most appropriate health care professionals to obtain a BPMH due to their high level of accuracy [8–13]. However, as a time and resource efficient alternative, studies have investigated the employment of pharmacy students and technicians in the process [4, 14–22]. There is extensive literature to support pharmacy technicians obtaining BPMHs for patients [4]. However, anecdotally, in Australia, pharmacy technicians have many competing duties, and are not trained across all Australian hospitals to obtain BPMHs. Therefore, an alternative, such as pharmacy students, represent a viable option to enhance medication continuity upon transition of care into hospital. Consensus documents agree that pharmacy students should be able to take a patient’s BPMH, as it is considered a core Entrustable Professional Activity (EPA) by the America Association of Colleges of Pharmacy [23]. Such core EPAs are discrete, essential activities and tasks that all new pharmacy graduates must be able to perform without direct supervision upon entering practice or postgraduate training [23].

A review of the literature has suggested that pharmacy-students are able to obtain an accurate BPMH, which is based on comparisons with non-pharmacy healthcare professionals [4]. Current literature establishes the ability of pharmacy students to undertake the entirety of the medication reconciliation process [14–17, 19, 20]. However, there are no studies directly comparing the quality of BPMHs obtained by pharmacy students compared to pharmacists [18]. To our knowledge, this is the first study evaluating the accuracy of pharmacy-student obtained BPMHs to pharmacist-obtained BPMHs.

### Aim

The primary aim of this study was to evaluate the proportion of patients who have an accurate BPMH from the pharmacy student-obtained BPMH compared to the pharmacist-obtained BPMH. The secondary objectives were to characterise the number, type, and severity of any deviations; and risk-factors associated with those deviations.

### Ethics approval

Ethics approval was obtained from Sydney Local Health District Ethics Review Committee (2019/ETH07525) as a quality improvement project of existing services, hence patient consent was not required. Approval was granted on 19th September 2018.

## Method

### Study design and setting

This was a prospective observational study from September 28th to November 18th, 2021, across two tertiary teaching hospitals, with approximately 900 and 750 beds, in the state of New South Wales, Australia.

### Participants

Final-year pharmacy students undertaking either a Bachelor or Master of Pharmacy degree in Australia were invited to participate in this study. The final-year students in this study were in their pre-internship training year. They are required to complete an additional year of internship training prior to licensure. Thus, pharmacy students in this study were earlier in their career trajectory than final-year pharmacy students in North America, where the internship is usually part of the pharmacy degree.

### Training

The students were taught how to obtain a BPMH from their host university as part of the EPAs in their regular curriculum, which they practiced regularly during their weekly tutorials. This training was based on guidelines published by the Australian Commission on the medication reconciliation process [7]. Students participating in the study received additional training on how to select a patient; how to best obtain a medication history using two sources (the first is to be an administration source, such as the patient or carer; and the second is to be a supplier source, such as the community pharmacy or GP); and how to document the medication history using the electronic medical records system (Powerchart, Cerner Millennium, Missouri, U.S.).

Students were informed that all adult patients taking five or more prescription-medications should be prioritised and considered eligible for this study. However, patients would be excluded if they had an altered mental or cognitive status (e.g., comatose patient) and no available carer to provide information; or the patient/carer primarily responsible for the medications cannot speak English; or the patient is from a community facility (e.g., nursing home) or has been transferred from a different healthcare facility (e.g., transfer from another hospital).

Students were allocated to a hospital site and completed one 8-h shift per week for 8 weeks. The students worked in pairs and were observed by the ward pharmacist who was responsible for supplementing the student training through individual feedback. The training process involved the ward pharmacist completing the EPA Assessment Tool form (see supplementary material A) to ensure competency, prior to the student pair obtaining medication histories without direct supervision. The form was adapted from a draft template set out by Pharmacy in South Australia [24]. The investigating pharmacist only recruited patients from a given pair of students once they were deemed competent to work independently based on the EPA Assessment Tool.

During the training process it was identified that each ward pharmacist trained their allocated students slightly differently regarding the documentation of combination medications. For example, for Janumet (metformin/sitagliptin), some students were trained to document the brand and others were trained to document each ingredient separately. The latter option is due to the limited availability of combination tablets in the hospital. Consequently, to standardise the medication count, the research team counted each ingredient for combination products for all patients, i.e., Janumet was counted as two medications.

### Data collection

An investigating pharmacist independently obtained a BPMH from patients within the 24 h after the student BPMH was obtained. Medication deviations were defined as any differences between the student-obtained and pharmacist-obtained BPMH. These deviations were initially categorised according to type, as per the medication discrepancy taxonomy (MedTax) [25]. This MedTax categorises deviations (or discrepancies) into two main groups: drug mismatch or drug partial match [25]. The drug mismatch subgroups include, but are not limited to, medication omission (i.e., unintentionally missing from the BPMH) or medication commission (i.e., incorrectly added to the BPMH). The drug partially matched subgroups include, but are not limited to, incorrect regimen, including route, dose, or frequency [25]. These deviations were then assessed based on their potential consequence, as either insignificant, minor, moderate, major or catastrophic according to risk classification published by The Society of Hospital Pharmacists of Australia (SHPA) [26]. The SHPA defined each of the risks as follows: (1) insignificant: no harm or injuries, low financial loss; (2) minor: minor injuries, minor treatment required, no increased length of stay or re-admission, minor financial loss; (3) moderate: major temporary injury, increased length of stay or re-admission, cancellation or delay in planned treatment/procedure. Potential for financial loss; (4) major: major permanent injury, increased length of stay or re-admission, morbidity at discharge, potential for significant financial loss; and (5) catastrophic: death, large financial loss and/or threat to good will/good name [26]. Please see supplementary material B for examples of medication deviations.

Medications with either no deviations or a deviation consequence considered insignificant, or minor were classified as ‘no-or-low risk’; and those with a moderate, major, or catastrophic consequence were classified as ‘high risk’. An accurate BPMH was defined as only having 'no-or-low risk’ medication deviations. The process of classifying each deviation was completed by two hospital pharmacists, who have at least five years of clinical experience, and any disagreements were discussed with a third senior academic clinical pharmacist. Final decisions were decided by consensus. If a patient had more than one medication deviation, the patient was categorised according to the medication deviation with the greatest consequence.

Student data collected included previous BPMH experience (the student was considered experienced if they had prior experience obtaining a BPMH from a patient in a hospital setting as a part of experiential education or as a hospital employee); degree type (Bachelors or Masters); public hospital pharmacy experience; and community pharmacy experience. The patient demographics collected were age, gender, hospital site, admitting clinical specialty, type of admission (planned or emergency) and Charlson-Comorbidity Index (CCI) score. The BPMH-related variables collected were the student pair that obtained the BPMH, and whether the student used two different types of information sources. The two types of sources were (1) a source that supplies the medication (e.g., general practitioner/specialist or community pharmacy) and (2) a source that administers the medication (e.g., patient, carer, or dose administration aid). Medication-related variables included the category and number of medications identified. The category was defined according to the Anatomical Therapeutic Chemical (ATC) classification [27]; and the medications were all counted and recorded by individual generic drug (for example, the combination tablet of sitagliptin and metformin counted as two medications, not one), and were classified as either regular prescription, regular non-prescription (this included over-the-counter medications and vitamins taken on a regular schedule), when-required prescription and when-required non-prescription.

### Sample size

As this was an observational study, we included every patient that received a BPMH by pharmacy students for the duration of the program. Only a limited number of students could be trained due to the COVID-19 pandemic. It was expected that less BPMHs would be obtained compared to prior years. Therefore, all student-obtained BPMHs were included and no formal sample size calculation was performed.

### Statistical analysis

Descriptive statistics were used to report all quantitative variables. Data are presented as mean (standard deviation) for continuous variables and percentages for categorical variables. The primary outcome measure was if a patient had a no-or-low risk deviation as previously defined. Thus, patients were dichotomized as those with no-or-low-risk deviations versus those with high-risk deviations for the primary analysis. Patient demographics were compared between the two categories using the Fishers exact test for categorical data. All patient data were complete, hence there were no missing data in this study.

Analyses were also completed at the medication level with all data tabulated comparing medication deviations with no-or-low risk to high-risk. These medication deviations were classified according to MedTax (omission, commission, and partial match), and by ATC categories.

A multivariable mixed effects logistic regression was conducted, with students assigned as the random effect. The dependent variable was if a BPMH had a high-risk medication deviation. Associations were examined between the dependent variable and the following independent variables: total number of medications, age, CCI score, hospital site, number of BPMH sources, and pharmacy student-related variables (degree type, community pharmacy experience, and previous experience obtaining BPMHs in a hospital setting). Adjusted odds ratios of independent variables were reported. For all analyses, a *p*-value of less than or equal to 0.05 was considered significant. Statistical analyses were computed using STATA (version 15, College Station, TX), and SPSS (version 28, IBM, Chicago, IL).

## Results

### Student demographics

Twelve final-year pharmacy students were trained to obtain BPMHs in 2 tertiary hospitals and one student withdrew from the study. Hence, 10 students completed BPMHs as pairs and one student performed all BPMHs without a partner. Of the final 11 students who completed the study, 8 were female (73%), 7 (64%) were undertaking a Bachelor of Pharmacy degree, 7 (64%) were working in a community pharmacy and none of the students had employment experience in a public hospital pharmacy. In addition, 6 (55%) indicated that they had prior experience to obtaining a BPMH in a hospital setting. On average, each student pair completed 15 BPMHs (SD = 1.56) over the 8-week period.

### Patient demographics

In total, 91 patients had a BPMH completed by the pharmacy students. Their mean age was 77 (SD = 15) years and 50.5% were male. A majority of patients’ admissions were an emergency (89.0%) and were not surgery-related (76.9%). As an indication of medical-history complexity, the mean CCI score was 6 (SD = 3). On average, patients reported taking 13 (SD = 5) home medications upon admission. This represented an average of 8 (SD = 5) regular prescription medications; 3 (SD = 2) regular non-prescription medications; 1 (SD = 1) when-required prescription medications; and 2 (SD = 1) when-required non-prescription medications. A similar number of patients were included from each site (54% and 46%). The difference in patient demographics between those with no-or-low and high-risk medication deviation consequence can be found in Table 1 in Supplementary material.

### Primary objective: BPMH discrepancies

In total, 71.4% (n = 65/91) of student-obtained BPMHs were considered accurate. Of these BPMHs 3 (3.3%) had no deviations, 35 (38.5%) had insignificant deviations, and 27 (29.7%) had minor deviations. Of the student-obtained BPMHs that were not accurate (n = 26/91), 16 (17.6%) had a moderate deviation and 10 (11%) had a major deviation.

### Secondary objective: medication discrepancies

There was a total of 1170 medications documented across all 91 BPMHs. This included 685 (58.5%) regular prescription medications; 266 (22.7%) regular non-prescription medications; 159 (13.6%) ‘when-required’ non-prescription medications; and 60 (5.1%) ‘when required’ prescription medications. The student-obtained medication histories had 439 deviations (37.5%, n = 1,170) from the medications documented. Hence, the students made an average of 5 (SD = 3.5) medication deviations per patient. Of the student-related deviations (n = 439), the medication types involved were regular prescription (37.4%, n = 164/439); regular non-prescription (25.3%, n = 111/439); when-required non-prescription (29.8%, n = 131/439); and when-required prescription (7.5%, n = 33/439). The MedTax categorisation of these medication deviations was drug omission (50.3%, n = 221/439); drug partially matched (33.0%, n = 145/439); or drug commission (16.6%, n = 73/439).

The risk ratings of the 439 medication deviations were as follows: insignificant (281, 64.0%), minor (106, 24.1%), moderate (39, 8.9%), major (13, 3.0%), or catastrophic (0, 0%). Thus, 88.2% (n = 387/439) were deemed no-or-low risk and 11.8% (n = 52/439) were high risk. Overall, 95.6% (n = 1118/1170) of medications documented were of no-or-low risk deviations, and 4.4% (n = 52/1170) were high-risk. These 52 high-risk medication deviations were across 28.6% patients (n = 26/91). Of the 387 no-or-low-risk medication deviations, a majority (38.5%, n = 149/387) were from the ‘Alimentary tract and metabolism’ class (110 out of the 149 were due to vitamins/minerals and drugs for constipation). Of the 52 high-risk medication deviations, all were regular prescription medications, and a majority were from both the ‘Alimentary tract and metabolism’ class (25%, n = 13/52, 8 out of the 13 were due to drugs in diabetes) and ‘Nervous system’ (25%, n = 13/52, 8 out of the 13 were due to Parkinson drugs). Furthermore, these high-risk medication deviations were significantly associated with ‘blood and blood forming organs’ medications (no-or-low risk 47.6% vs high-risk 52.4%, *p* < 0.001).

### Factors associated with patients having an accurate BPMH

In the mixed effects multivariable logistic regression, students were more likely to obtain an accurate BPMH in older patients (OR 1.04; 95% CI 1.03–1.06; *p* < 0.001), patients taking fewer medications (OR 0.85; 95% CI 0.75–0.97; *p* = 0.02) and when students used two source types (administration and supplier) to compile the BPMH (OR 1.65; 95% CI 1.09–2.50; *p* = 0.02).

## Discussion

The key finding of this study is that 71.4% of BPMHs obtained by pharmacy students were accurate. This should be viewed in the context of the deviation rate in practice. For instance, Canning et al. examined the accuracy of pharmacist-obtained medication histories when checked for accuracy by another pharmacist [28]. Of the 99 BPMHs obtained, 85% of pharmacist-obtained BPMHs had no-or-low risk discrepancies, using a similar definition as our study [29]. This confirms that pharmacists are not immune to errors in BPMHs. However, there are two noteworthy points. First, an Australian study identified that only half the patients had a medication reconciliation completed within 24 h of admission [30]. This highlights that there is a dire need for additional resourcing to support pharmacy-led medication continuity and patient safety during transition of care. Patient safety would be better supported with students obtaining BPMHs with a 71.4% accuracy rate, rather than patients receiving no care. Additionally, although the pharmacy student accuracy rate is 71.4%, literature confirms that pharmacy students appear to be more accurate than doctors (61.4%) [31] and nurses (52%) [21]. Hence, pharmacy students may be incorporated into the BPMHs process in hospital with severe staffing constraints to reduce the burden on other healthcare professionals. Second, it is noteworthy that based on this study, the University of Sydney has adjusted its program so that students do not document the BPMH. The student role has become more conducive to pharmacists. In the major metropolitan hospitals, where staffing is adequate, students are now required to undertake the time-consuming processes to assist the pharmacist. The primary role of students now, is to obtain a preliminary list of medications and obtain written verification from a second source. The verification using a second source is quite time-consuming. For example, using a community pharmacy as a second source involves contacting the pharmacy, requesting a dispensing history to be faxed and following up any delays. Having pharmacy students undertake this process is time-efficient and provides pharmacists with the necessary support to optimise medication continuity across transition of care.

Our study also identified that the accuracy of BPMHs is significantly increased if the student used two types of information sources. In Australia, taking a BPMH involves contacting two sources, but does not restrict the types of sources used (supplier and administration source) [7, 28, 29]. The use of two sources for a BPMH is not consistent in literature [16, 20–22, 31], with only a few studies mandating a second source [14, 18, 19]. Our study confirms the international Delphi study that a BPMH should be based on at least two types of sources (from an administration and a written source) [32].

Our study identified that high-risk medication deviations were significantly associated with ‘blood and blood forming organs’ medications, which is consistent with literature [33, 34]. Similarly, to other studies, the most common discrepancy type was drug omission [4, 14, 15, 17, 20, 34, 35]. Our study also identified that no-or-low-risk medication deviations were predominantly associated with non-prescription medications (e.g., vitamins, and “when needed” medications), which implies a difference between the pharmacist and the student’s BPMH questioning process. This is consistent with literature, with studies reporting that non-prescription medications had the most discrepancies [15, 19]. Additional focus in these areas could be incorporated in future student training.

There were no student factors (e.g., degree type, community pharmacy experience and BPMH experience) significantly associated with a patient having a high-risk discrepancy. Similarly, currently literature reports that there are no student factors associated with medications documented with discrepancies [16, 31].

### Limitations

First, our results should be extrapolated with caution to other countries due to differences in the level of student training. Second, the risk level of a medication deviation was based on subjective assessment. However, two pharmacists completed this task to reduce bias. A healthcare professional with a medical perspective could have further improved the assessment of deviations. Third, the results could be influenced by the training process used for students. We anticipate that by improving the training process accuracy of BPMHs would increase. Fourth, our study only included 11 students, and our results may not be generalisable to all students. Fifth, students were allocated in pairs to provide a supportive learning environment where they could assist one another. However, further research may identify when this is no longer required. Six, only one pharmacist was responsible for the accuracy check. Although this was advantageous in terms of consistency, it also means that the results were dependent on the accuracy of that pharmacist who has extensive experience obtaining BPMHs. Finally, we did not assess the economic or clinical impact of the medication deviations on patient outcomes (e.g., readmission rates) as this was outside the scope of the study.

## Conclusion

It is suitable for pharmacy students to be incorporated into the BPMHs process and for student-obtained BPMHs to be verified for accuracy by a pharmacist.

## Supplementary Information

Below is the link to the electronic supplementary material.

Supplementary file1 (DOCX 32 kb)
