# Supplementary material for: Accuracy of best possible medication histories by pharmacy students: an observational study
Source: Int J Clin Pharm. 2022 Dec 14;45(2):414–20. doi: 10.1007/s11096-022-01516-2 (PMC9749631; doi:10.1007/s11096-022-01516-2)
Supplement: Supplementary file 1 — Supplementary file1 (DOCX 32 kb) [file 11096_2022_1516_MOESM1_ESM.docx]

**SUPPLEMENTARY MATERIALS**

**Supplementary material A –** SYDNEY LOCAL HEALTH DISTRICT (SLHD) PHARMACY ENTRUSTABLE PROFESSIONAL ACTIVITY TOOL: BEST POSSIBLE MEDICATION HISTORY (BPMH) & RECONCILIATION

| **SLHD PHARMACY ENTRUSTABLE PROFESSIONAL ACTIVITY TOOL:**  **BEST POSSIBLE MEDICATION HISTORY (BPMH) & RECONCILIATION** | | **Comments** | **Scoring** | | |
| --- | --- | --- | --- | --- | --- |
| **1.1 Electronic Medical Record (eMR)** | - Student is able to navigate the eMR   - Students to review relevant information in the eMR before meeting with the patient |  | **Y** | **N** | **N/A** |
| **1.2 Relevant patient background** | - Supervising pharmacist to preselect a potential patient - Students to obtain and contextualise patient information such as residence (e.g. rural or nursing home), age, gender, height/weight, pregnancy, immunisation status, smoking status, previous medical history, working diagnosis, relevant laboratory findings, Culturally and Linguistically diverse status |  | **Y** | **N** | **N/A** |
| **1.3 On arrival to the ward** | - Student speaks to Nurse-Unit-Manager/Nurse-In-Charge to ensure a suitable patient has been selected |  | **Y** | **N** | **N/A** |
| **1.4 Introduction to consultation & Patient Consent** | - Student introduces themselves to the patient   - Confirm patient identity using 2 identifiers (e.g. full name and DOB)   - Describes what they are going to do and request patient consent   - Provide clear introduction to the consultation (confirm time is convenient, establish rapport) |  | **Y** | **N** | **N/A** |
| **1.5 BPMH questioning & interviewing technique** | - Communication is appropriate   - Establish the most appropriate person to obtain medication history from   - Use appropriate questioning to obtain relevant information from the patient (succinct and logical)   - Appropriate verbal & non-verbal language (including tone)   - Open & closed questions   - Summarise the interview |  | **Y** | **N** | **N/A** |
| **1.6 BPMH content & MHR documentation** | - BPMH   - Ask about prescription, non-prescription and complementary medicines   - Ask about immunisation (if appropriate)   - Ask about recently ceased or changed medicines - Confirm patients understanding of their medicines e.g. warfarin - Correctly documented (using generic names (add trade names where appropriate e.g. warfarin, slow-release opioids); dose, frequencies etc are correct) - Appropriate allergy/ adverse drug reaction (ADR) history and documentation |  | **Y** | **N** | **N/A** |

Abbreviations: DOB: date of birth; MHR: medical health record

| **SYDNEY LOCAL HEALTH DISTRICT (SLHD) PHARMACY ENTRUSTABLE PROFESSIONAL ACTIVITY TOOL:**  **BEST POSSIBLE MEDICATION HISTORY (BPMH) & RECONCILIATION** | | | **Comments** | **Scoring** | | | | |
| --- | --- | --- | --- | --- | --- | --- | --- | --- |
| **1.7 Confirmation** | - Confirm medication history with at least one secondary source to ensure accuracy and completeness (if appropriate). Sources documented. - Record community pharmacy name and contact details (if utilised) | |  | **Y** | **N** | | **N/A** | |
| **1.8 BPMH documentation & electronic medication reconciliation** | - Reconcile:   - Currently prescribed medicines with those taken prior to presentation   - Medicines with medical conditions - Complete MHR Form, document history via AdHoc intervention form and document in progress notes using SOAP (Subjective; Objective; Assessment; Plan) format. | |  | **Y** | **N** | | **N/A** | |
| **1.9 Discussion with ward pharmacist** | - Student understands the limits of their knowledge and that they can seek advice - Handover to ward pharmacist is in a structured manner - Identify the need to obtain further information or advice - Recognise limits of personal knowledge and/or ability to interpret information | |  | **Y** | **N** | | **N/A** | |
| **WARD PHARMACIST ASSESSMENT** | | | | | | | | |
| **Please circle whether students performed the patient interview at the bedside or via telephone** | | | Face-to-face interview | | | Via telephone | |  |
| Students communicated well and there was no critical information left out, **i.e. Proceed with minimal supervision** | | | | **Y** | **N** | |  | |
| Students performed most tasks well however either a critical step left out or needs to improve before working with minimal supervision, **i.e. Additional BPMH observed or Action Plan (see below) before a subsequent observation** | | | | **Y** | **N** | | **N/A** | |
| Student pair is unable to independently perform BPMH, **i.e. Multiple steps left out & poor communication. Contact University for advice** | | | | **Y** | **N** | | **N/A** | |
| **ACTION PLAN** | | | | | | | | |
| **Student pair (name & signature)** | |  | | | | | | |
| **Pharmacist (name & signature)** | |  | | | | | | |
| **Date** | |  | | | | | | |

**Supplementary material B**

| Potential consequence of deviation | Example |
| --- | --- |
| Insignificant | Drug omission of sorbolene cream |
| Minor | Omission of day of dulaglutide weekly subcutaneous injection |
| Moderate | Omission of antihypertensive medication |
| Major | Commission of buprenorphine topical patch |
| Catastrophic | Commission of medication to which a patient has an anaphylactic reaction |

**TABLES**

**Table 1. Patient demographics (n=91)**

| Patient Demographics | | Deviation risk of Best Possible Medication Histories | | |  | |  |
| --- | --- | --- | --- | --- | --- | --- | --- |
| Age, n (%) |  | No-or-low risk, n=65 (%) | High risk, n=26 (%) | Total, n (%) | | p-value | |
|  | <65 years | 10 (15.4) | 4 (15.4) | 14 (15.4) | | 0.90 | |
|  | 65-75 years | 15 (23.1) | 6 (23.1) | 21 (23.1) | |  |  |
|  | 76-85 years | 22 (33.8) | 7 (26.9) | 29 (31.9) | |  |  |
|  | >85 years | 18 (27.7) | 9 (34.6) | 27 (29.7) | |  |  |
| Sex, n (%) |  |  |  |  | |  | |
|  | Female | 34 (52.3) | 11 (42.3) | 45 (49.5) | | 0.49 | |
| Hospital site, n (%) |  |  |  |  | |  | |
|  | Hospital site A | 40 (61.5) | 9 (34.6) | 49 (53.9) | | 0.04 | |
| Type of admission, n (%) |  |  |  |  | |  | |
|  | Emergency | 60 (92.3) | 23 (88.5) | 81 (89.0) | | 0.14 | |
| Surgery-related admission, n (%) |  |  |  |  | |  | |
|  | No | 50 (76.9) | 20 (76.9) | 70 (76.9) | | 1.00 | |
| Charlson Comorbidity Index Score, mean (STD) |  | 5.79 (3.22) | 6.95 (2.74) | 6.27 (3.07) | |  | |
| Time student-obtained BPMH from point of admission |  |  |  |  | |  | |
|  | <24 hours | 16 (24.6) | 5 (19.2) | 21 (23.0) | | 0.30 | |
|  | 24-48 hours | 13 (20) | 3 (11.5) | 16 (17.6) | |  |  |
|  | 49-72 hours | 10 (15.4) | 2 (7.7) | 12 (13.2) | |  |  |
|  | >72 hours | 26 (40) | 16 (61.5) | 42 (46.2) | |  |  |

Abbreviations: STD: Standard deviation

**Table 2. Medication deviation details from student-obtained best possible medication histories (n=439/1170)**

| Medication Details | | Medication Discrepancy Type of: | | |  |
| --- | --- | --- | --- | --- | --- |
|  |  | No-or-low risk, n (%) | High risk, n (%) | Total, n (%) | p-value |
| Number of medications recorded,  n |  |  |  |  |  |
|  | Regular prescription medication, n (%) | 633 (92.4) | 52 (7.6) | 685 (58.5) | N/A |
|  | Regular non-prescription medication, n (%) | 266 (100) | 0 (0) | 266 (22.7) |  |
|  | When-required prescription medication, n (%) | 60 (100) | 0 (0) | 60 (5.1) |  |
|  | When-required non-prescription medication, n (%) | 159 (100) | 0 (0) | 159 (13.6) |  |
|  | **Total** | 1118 (100) | 52 (100) | 1170 (100) |  |
| Number of medication deviations,  n |  |  |  |  |  |
|  | Regular prescription medication, n (%) | 112 (28.9) | 52 (100) | 164 (31.7) | <0.001 |
|  | Regular non-prescription medication, n (%) | 111 (28.7) | 0 (0.0) | 111 (25.3) | <0.001 |
|  | When-required prescription medication, n (%) | 33 (8.5) | 0 (0.0) | 33 (7.5) | 0.013 |
|  | When-required non-prescription medication, n (%) | 131 (33.9) | 0 (0.0) | 131 (29.8) | <0.001 |
|  | **Total** | 387 (100) | 52 (100) | 439 (100) |  |
| Medication deviation type, n |  |  |  |  |  |
|  | Drug omission, n (%) | 198 (51.2) | 23 (44.2) | 221 (50.3) | 0.38 |
|  | Drug commission, n (%) | 55 (14.2) | 18 (34.6) | 73 (16.6) | <0.001 |
|  | Drug partial match, n (%) | 134 (34.6) | 11 (21.2) | 145 (33.0) | 0.06 |
|  | **Total** | 387 (100) | 52 (100) | 439 (100) |  |
|  |  |  |  |  |  |
| Anatomical therapeutic chemical classification of medication deviations, n |  |  |  |  |  |
|  | Alimentary tract and metabolism, n (%) | 149 (38.5) | 13 (25.0) | 162 (36.9) | 0.04 |
|  | Blood and blood forming organs, n (%) | 10 (2.6) | 11 (21.2) | 21 (4.8) | <0.001 |
|  | Cardiovascular system, n (%) | 32 (8.3) | 5 (9.6) | 37 (8.4) | 0.45 |
|  | Dermatologicals, n (%) | 26 (6.7) | 0 (0.0) | 26 (5.9) | - |
|  | Genito urinary system and sex hormones, n (%) | 5 (1.3) | 1 (1.9) | 6 (1.4) | 0.53 |
|  | Systemic hormonal preparations, excluding sex hormones and insulins, n (%) | 2 (0.5) | 1 (1.9) | 3 (0.7) | 0.32 |
|  | Anti-infectives for systemic use, n (%) | 5 (1.3) | 2 (3.8) | 7 (1.6) | 0.20 |
|  | Antineoplastic and immunomodulating agents, n (%) | 5 (1.3) | 3 (5.8) | 8 (1.8) | 0.06 |
|  | Musculoskeletal system, n (%) | 15 (28.8) | 2 (3.8) | 17 (3.9) | 0.67 |
|  | Nervous system, n (%) | 84 (21.7) | 13 (25.0) | 97 (22.1) | 0.35 |
|  | Respiratory system, n (%) | 32 (8.3) | 0 (0.0) | 32 (7.3) | - |
|  | Sensory organs, n (%) | 22 (5.7) | 0 (0.0) | 22 (5.0) | - |
|  | Various, n (%) | 0 (0.0) | 1 (1.9) | 1 (0.2) | 0.12 |
|  | **Total** | 387 (100) | 52 (100) | 439 (100) |  |

**Table 3. Mixed effects logistic regression clustered for student pairs to predict Best Possible Medication History accuracy with no-or-low-risk medication deviations**

|  | Odds Ratio (95% CI) | P-VALUE |
| --- | --- | --- |
| Type of *‘*Best Possible Medication History’ sources |  |  |
| One source type | Reference | Reference |
| Two source types | 1.65 (1.09 – 2.50) | 0.02 |
| Student degree type |  |  |
| Bachelor of Pharmacy | Reference | Reference |
| Master of Pharmacy | 2.31 (0.06 – 86.83) | 0.65 |
| Community pharmacy experience |  |  |
| No | Reference | Reference |
| Yes | 0.30 (0.04 – 2.62) | 0.27 |
| Student had previous experience taking a best possible medication history |  |  |
| No | Reference | Reference |
| Yes | 0.71 (0.08 – 6.45) | 0.76 |
| Hospital site |  |  |
| Site A | Reference | Reference |
| Site B | 0.23 (0.05 – 1.02) | 0.05 |
| Charlson Comorbidity Index score | 0.93 (0.76 – 1.15) | 0.51 |
| Patient age | 1.04 (1.03 – 1.06) | <0.001 |
| Total number of medications | 0.85 (0.75 – 0.97) | 0.02 |
